# Evaluation of the Phytochemical and Pharmacological Potential of Taif’s Rose (*Rosa damascena* Mill var. *trigintipetala*) for Possible Recycling of Pruning Wastes

**DOI:** 10.3390/life12020273

**Published:** 2022-02-12

**Authors:** Tarek M. Galal, Hatim M. Al-Yasi, Mustafa A. Fawzy, Tharwat G. Abdelkader, Reham Z. Hamza, Ebrahem M. Eid, Esmat F. Ali

**Affiliations:** 1 Department of Biology, College of Sciences, Taif University, P.O. Box 11099, Taif 21944, Saudi Arabia; t.aboseree@tu.edu.sa (T.M.G.); h.alyasi@tu.edu.sa (H.M.A.-Y.); mafawzy@tu.edu.sa (M.A.F.); t.abdelkader@tu.edu.sa (T.G.A.); reham.z@tu.edu.sa (R.Z.H.); 2 Biology Department, College of Science, King Khalid University, P.O. Box 9004, Abha 61321, Saudi Arabia; ebrahem.eid@sci.kfs.edu.eg; 3 Botany Department, Faculty of Science, Kafrelsheikh University, Kafr El-Sheikh 33516, Egypt

**Keywords:** Damask rose, pruning wastes, phytochemicals, biological activity, recycling

## Abstract

This study investigated the phytochemical contents of Taif’s rose pruning wastes and their potential application as phytomedicine, thereby practicing a waste-recycling perspective. In the Al-Shafa highland, four Taif rose farms of various ages were chosen for gathering the pruning wastes (leaves and stems) for phytochemical and pharmacological studies. The leaves and stems included significant amounts of carbohydrates, cardiac glycosides, alkaloids, flavonoids, and other phenolic compounds. The cardiac glycoside and flavonoid contents were higher in Taif rose stems, while the phenolic and alkaloid contents were higher in the plant leaves. Cardiovascular glycosides (2.98–5.69 mg g^−1^), phenolics (3.14–12.41 mg GAE g^−1^), flavonoids (5.09–9.33 mg RUE g ^−1^), and alkaloids (3.22–10.96 mg AE g^−1^) were among the phytoconstituents found in rose tissues. According to the HPLC analysis of the phenolic compounds, Taif’s rose contains flavonoid components such as luteolin, apigenin, quercetin, rutin, kaempferol, and chrysoeriol; phenolics such as ellagic acid, catechol, resorcinol, gallic acid, and phloroglucinol; alkaloids such as berbamine, jatrorrhizine, palmatine, reticuline, isocorydine, and boldine. Warm water extract was highly effective against *Bacillus subtilis*, *Escherichia coli*, and *Proteus vulgaris*, whereas methanol and cold water extracts were moderately effective against *Aspergillus fumigatus* and *Candida albicans*. The study’s findings suggested that Taif’s rose wastes could be used for varied medical purposes.

## 1. Introduction

*Rosa damascena* Mill. var. *trigintipetala* (Taif’s rose or Damask rose), a Rosaceae plant, is one of the most important commercial crops farmed due to the high value of its essential oils worldwide [1]. It is a tall shrub that can reach 2.5 meters in height and blooms once a year (in May–June), with a fully mature plant producing 500–600 flowers [2]. Taif’s rose grows in temperate and subtropical climates at elevations ranging from 300 to 2500 meters [3]. It is commercially grown in Saudi Arabia, Egypt, Turkey, Morocco, Bulgaria, Iran, France, China, and India, among other countries [4]. It is also one of the attractive and aromatic plants grown for use in the perfume, pharmaceutical, and food industries in numerous Taif governorate locations [5]. In contrast to the Bulgarian variety, the Saudi Arabia rose oils do not fully comply with the ISO 9842:2003 standard for rose oil, but they have a high olfactive potential [6]. Taif’s rose has been shown to have antioxidant, antidiabetic, anti-HIV, antibacterial, anti-inflammatory, and cardiotonic properties due to the presence of various phytochemical compounds such as alkaloids, phenolic acids, flavonoids, and other phenolic compounds [7,8,9].

In terms of the current state of the Taif rose, the governorate of Taif has approximately 860 farms ranging in size from large (1.0 ha) to small (0.03 ha) with most of them ranging from 0.3 to 0.7 ha. This variation in size may be due to the cultivation conditions of the Taif rose on mountain ridges and in wadi beds, which differs from the corresponding species worldwide. The wastes generated by these farms (>2700 ton) could be agriculturally produced from rose bush pruning and industrially created from the oil distillation process [10]. A tiny portion is used for vegetative propagation, but the majority is dried and burned, posing environmental issues such as air and soil pollution, as well as health risks to surrounding residents [11]. The output of Taif rose blooms, and thus the highest oil percentage, is closely related to trimming [12]. Pruning is carried out such that the lower branches get sufficient light to create food, changing growth phases to encourage new axillary and bloom buds, and removing disease dependent on the variety [1].

Thousands of plant species are employed in many traditional systems of medicine around the world and are recognized for their contributions to contemporary medicine, with some of them, such as *Brugmansia* and *Rosa* species, being used to treat cancer and cardiovascular problems [13,14]. The use of medicinal plants is a centuries-old tradition, and recent advances in contemporary therapies have boosted the use of natural products for a variety of maladies and disorders around the world [15]. Secondary metabolites have a variety of biological effects, and they provide the scientific foundation for many ancient civilizations’ uses of herbs in traditional medicine [16]. Phenolic compounds are widely distributed and the most abundant secondary metabolites in plants; they include flavonoids, alkaloids, and phenolic acids, which are involved in the defense against ultraviolet radiation or aggression by pathogens, parasites, and predators [17]. These secondary plant metabolites were investigated for their activity against cardiovascular and neurodegenerative diseases and cancer [17,18].

Pharmacological investigations have demonstrated that rose blooms of Taif provide a wide range of health benefits due to their high polyphenolic content [19]. According to Karkania et al. [20], *R. damascena* has potential antimicrobial activities against both Gram-negative and Gram-positive bacteria as well as fungi. Moreover, strong antimicrobial activity has been reported against different bacterial and fungal strains such as *Escherichia coli*, *Proteus vulgaris*, *Candida albicans*, and *Staphylococcus aureus* [21,22]. On a scientific level, empirical knowledge from folk medicine is becoming an increasingly important component of in vitro and in vivo studies, including preclinical and clinical trials. These studies investigated and explained the therapeutic efficacy of rose products and their ingredients, such as antidepressant effects, psychological relaxation, sexual dysfunction improvement, antioxidant, antimicrobial, antifungal, probiotic, and antipyretic effects, smooth muscle relaxation, lipid-lowering content, antiulcerogenic effects, and so on [4,23,24,25]. 

Because of the associated transportation, storage, and processing requirements, direct burning of agricultural biomass or trash is not cost-effective [20]. Furthermore, inappropriate agricultural waste storage pollutes the environment (soil, air, water, and sight) [26]. Several studies were carried out on the pharmacological activity of the essential oil of Taif’s rose [6,27], while, to the authors’ knowledge, no studies have been conducted on the recycling of its vegetative wastes. As a result, recycling and reusing these solid wastes for commercial purposes is extremely important and essential. As a result, the current study intends to investigate the phytochemical elements including cardiac glycosides, flavonoid, alkaloids, and other phenolic compounds, of Taif’s rose pruning wastes and their pharmacological potential as a phytomedicine. These compounds, in addition to having antioxidant properties, have several other specific biological actions in preventing and/or treating diseases.

## 2. Material and Methods

### 2.1. Plant Sampling

During December 2020, four Taif rose farms on the Al-Shafa highland, Taif Province, Saudi Arabia, were chosen to collect pruning wastes for prospective recycling in medical uses. Farms F1, F2, F3, and F4 had ages ranging from 10, 12, 20, and 4 years, respectively. At each farm, ten rose plants of various sizes were chosen to estimate the biomass of their fresh pruning wastes. Shrubs were pruned until they reached a height of 80–90 cm. Fresh wastes were collected and weighed to determine their fresh biomass (kg ha^−1^) by multiplying the average individual weight by the number of individuals per farm. Then, samples were left for air drying for about two weeks until constant weight.

### 2.2. Sample Preparation

For plant analysis, three composite samples (leaves and stems) of trimmed vegetative wastes were collected from each farm. Plant materials were rinsed in tap and distilled water, then air-dried at room temperature in the shade before being homogenized in a planetary high-energy mill with a hardened chromium steel vial.

### 2.3. Quality Analysis

Approximately 250 g sample of plant powder was shaken in 1000 mL ethanol for 24 hours on an orbital shaker at room temperature, and then the extract was filtered with Whatman No 1 filter paper. The filtrate was concentrated to dryness under reduced pressure at 40 °C through evaporator. The extract was stored between 2 and 8 °C for analysis of alkaloids, phenolic acids, flavonoids, and cardiac glycosides. HCl, NaCO_3_, ethanol, Baljet’s solution, picric acid, NaOH, AlCl_3_, methanol, Folin reagent, NaHCO_3_, formic acid, acetonitrile, glacial acetic acid, diethylamine, dimethyl sulfoxide, Ketoconazole, and Gentamicin were the used chemical reagents.

#### 2.3.1. Determination of Soluble Carbohydrates

According to Sadasivam and Manickam [28], the total soluble carbohydrates were calculated using the anthrone method. Approximately 100 mg of Taif’s rose powder was hydrolyzed in a boiling water bath for 3 hours with 5 mL of 2.5 N HCl. The acid digested sample was chilled to room temperature before adding sodium carbonate to neutralize it. Using distilled water, the final volume was diluted to 100 mL and centrifuged for 15 min at 5000 rpm. The total soluble carbohydrates were then determined by collecting the supernatant.

#### 2.3.2. Determination of Cardiac Glycosides

Solich et al. [29] and Tofighi et al. [30] used techniques to measure cardiac glycosides. To detect cardiac glycosides, a 10% ethanol extract was mixed with 10 mL newly prepared Baljet’s solution (95 mL of 1% picric acid + 5 mL of 10% NaOH). After an hour, the liquid was diluted with 20 mL distilled water, and the absorbance was measured with a spectrophotometer (CECIL CE 1021, Cecil Instruments Limited, Corston, UK) at 495 nm.

#### 2.3.3. Determination of Total Flavonoid Contents (TFC) 

Tofighi et al. [30] published procedures for calculating the TFC of vegetative pruning wastes. Ten milligrams of plant leaves were extracted under reflux (80 °C) for 60 min with a 20 mL water–ethanol solution 60% (*v*/*v*) (pH = 5.06). After cooling to room temperature, the extract was filtered, and the residue was extracted again under the same conditions. The hydroalcoholic extract and the re-extract were combined, and the volume was raised to 50 mL of water–ethanol solution at 60% (*v*/*v*) (stock solution). To bring the stock solution to volume, a part of it was transferred to a 10 mL volumetric flask and mixed with methanol (blank solution). A second aliquot of the stock solution was transferred to a new 10 mL volumetric flask, which was then filled with 2% AlCl_3_ and brought to volume with methanol (test solution). After 25 min, the absorbance of the test solution was measured at 430 nm against a blank solution.

The rutin content of the TFC herbal material was determined as the average of three determinations. The flavonoid content (mg g^−1^) of herbal material (adjusted for moisture content) was determined as follows: TFC herbal material = (TFC tested solution × 1.25 × 50)/(w-ld), where TFC test solution is the total concentration of flavonoids in the test solution (mg mL^−1^), 1.25 corresponds to the dilution factor, 50 is the volume of the stock solution (mL), “w” is the weight of herbal material (g), and “l d” is the loss on drying of herbal material.

#### 2.3.4. Determination of The Total Phenolic Compounds 

The concentration of phenolics in the plant ethanol extract was determined using a spectrophotometric method [30,31]. The reaction mixture consisted of 0.5 mL ethanol extract, 2.5 mL 10% Folin–Ciocalteu’s reagent mixed in water, and 2.5 mL 7.5% NaHCO_3_. The blank was made with 0.5 mL methanol, 2.5 mL 10 percent Folin–Ciocalteu’s reagent dissolved in water, and 2.5 mL 7.5 percent NaHCO_3_. The samples were then incubated in a thermostat for 45 min at 45 °C. A spectrophotometer was used to measure the absorbance at 765 nm (CECIL CE 1021, Cecil Instruments Limited, Corston, UK). The samples were made in triplicate for each assay, and the mean absorbance value was computed. The calibration curve for the standard gallic acid solution was created using the same method. The amount of phenolics was measured in milligrams of gallic acid equivalent (GAE) per gram of dry weight (DW).

#### 2.3.5. Estimation of Phenolic and Flavonoid Compounds Using HPLC 

High-performance liquid chromatography (HPLC) was used to estimate the flavonoid and phenolic compounds of Taif’s rose plants. HPLC-MS techniques are often used for the separation, identification, and quantitation of flavonoids, phenolic acids, and other phenolic compounds in plants. The HPLC-MS system (Agilent 1100: Agilent Corp., Palo Alto, Calif.) is composed of a quaternary pump, a photodiode-array detector, a U*v*/*v* is detector, and a single quadrupole MS detector with an ion source (ESI). Flavonoids were separated in 70 min using a gradient solvent system of 0.1% formic acid solution with a flow rate of 1.0 mL min^−1^, detected at 280 nm, and identified by ESI-MS [32]. Phenolic acid was separated in 60 min using a gradient mobile phase of water/acetonitrile/glacial acetic acid (980/20/5, *v*/*v*/*v*, pH 2.68) and acetonitrile/glacial acetic acid (1000/5, *v*/*v*), with a flow rate of 0.8 mL min^−1^ and detection at 325 nm [33]. Moreover, alkaloids were analyzed by HPLC (0 min, 80:20 (A–B); 5 min, 80:20; 20 min, 60:40; 25 min, 0:100) using 0.2% diethylamine and 0.16% formic acid as solvent system A, and 0.2% diethylamine and 0.16% formic acid in acetonitrile as solvent system B. The column used was the GraceSmart RP18 (Grace Vydac, Hesperia, CA, USA), 5 µm, 250 mm × 4.6 mm with a flow rate of 1.0 mL min^−1^. The peaks were detected at 226 nm.

### 2.4. Biological Activity

#### 2.4.1. Preparation of Extracts

Approximately 250 grams of plant powder was steeped in 1.5 liters of 95% ethanol and methanol and boiled in cold (approximate room temperature) and warm (50 °C) water at room temperature for 5 days. The combination was blended daily to provide a consistent infusion. The extract was filtered using Whatman filter paper No. 1 after 5 days. A rotary evaporator at 60 °C was used to dry the filtrate. The dried extract was kept at −20 °C in sterile glass vials until use [34].

#### 2.4.2. Microorganisms Used

The following microorganisms were obtained from Al-Azhar University, Faculty of Science: gram-positive bacteria (*Bacillus subtilis*), gram-negative bacteria (*Escherichia coli* and *Proteus vulgaris*), and fungal strains (*Aspergillus fumigatus* and *Candida albicans*). The bacterial and fungal strains were cultured in nutrient agar and malt extract, respectively. 

#### 2.4.3. In Vitro Evaluation of the Antimicrobial Activity

An antimicrobial susceptibility test was performed using the agar disc well diffusion method [35] with some modifications. The diameter of inhibitory zones was used to measure antimicrobial activity. Plant extracts were tested against bacterial isolates as antimicrobial agents. On the surface media, inoculum suspensions of all bacterial and fungal isolates were distributed. Using a 6 mm Cork borer, holes (diameter 6 mm) were drilled into the media. The dried plant extracts were treated in dimethyl sulfoxide (DMSO) to make a 10 mg mL^−1^ final extract. Each plate’s well was filled with 100 µL of plant extract. The inoculated agar plates were incubated for 24 hours at 37 °C for bacterial growth and 48 hours at 28 °C for fungal growth. After 24–48 hours of incubation, inhibition zones caused by active extract components were measured. The studies were performed in triplicate, and the inhibition zone was assessed using a standard scale [36]. Ketoconazole antibiotic (MIC = 100 µg mL^−1^) was used as control treatment for fungi, while gentamicin (MIC = 4 µg mL^−1^) was used for the bacteria.

### 2.5. Statistical Analysis

The differences in plant’s chemical constituents in separate farms were analyzed by one-way analysis of variance (ANOVA I), using SPSS software (version 22), after the data were checked for normality [37]. When there were substantial variations among the farms, a post-hoc test was used (Duncan’s test).

## 3. Results 

### 3.1. Biomass of Pruning Wastes 

Taif’s rose was pruned in December after the rainy season, where the fresh biomass (FW: fresh weight) of the pruning wastes increased with increasing plant age (Table 1). The highest biomass (5.2 t FW ha^−1^) was recorded at the oldest farm (F3), while the lowest (1.3 t FW ha^−1^) was recorded at the youngest farm (F4). The average biomass produced from the different farms was 3.2 t FW ha^−1^.

### 3.2. Chemical Constituents

The analysis of total soluble carbohydrates (in dry weight) indicated significant variation (*p* < 0.001) in their tissue contents among the different study farms (Table 2). It was found that Taif’s rose leaves had higher carbohydrate contents than the stems, where the highest content (3.05%) was recorded in the leaves of F3 plants, while the lowest (0.78%) was found in F1 stems. The phytochemical screening of the ethanolic extract of Taif’s rose detected significant variations (*p* < 0.001) in the contents of cardiac glycosides, flavonoids, alkaloids, and phenolic compounds among the study farms (Table 2). Notably, Taif’s rose stems had higher cardiac glycoside and flavonoid contents, while leaves had higher phenolic and alkaloid contents. The plant leaves from F3 had the highest phenolic content (12.41 mg GAE g^−1^) but the lowest cardiac glycoside and flavonoid contents (2.98 mg securiaside g^−1^ and 5.09 mg RUE g^−1^). In addition, the stems of the F4 plants had the highest cardiac glycosides (5.69 mg securiaside g^−1^) but the lowest phenolic and alkaloid contents (3.14 mg GAE g^−1^ and 3.22 mg AE g^−1^). Moreover, the highest flavonoids (9.33 mg RUE g^-1^) were recorded in the stems of the F2 plants, while the highest alkaloid content (10.96 mg AE g^−1^) was found in the leaves of the F1 plants.

### 3.3. HPLC of Phytochemical Compounds

#### 3.3.1. Phenolic Compounds

**Figure 1:**
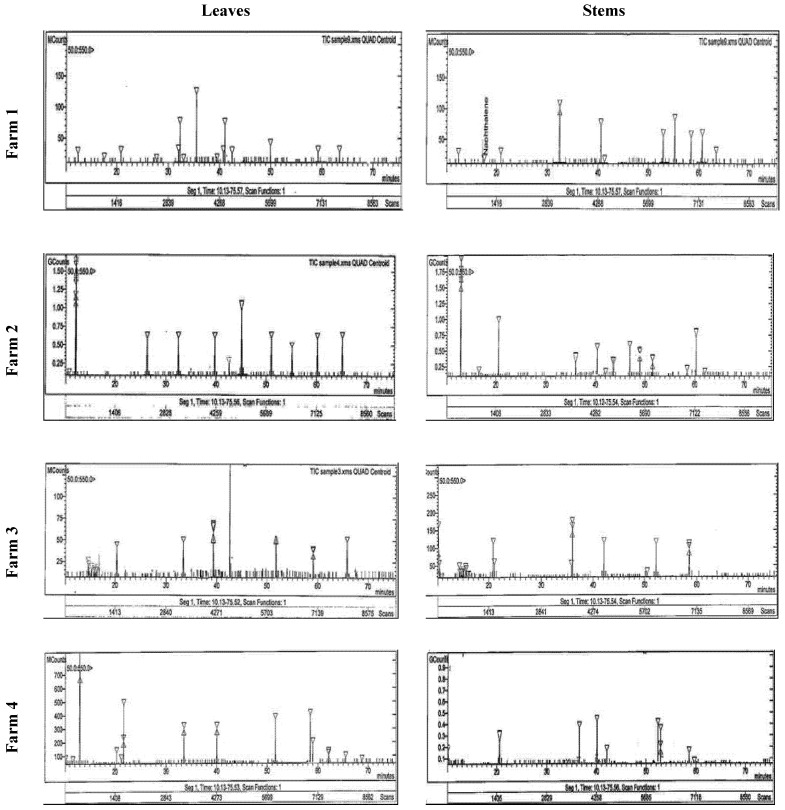
HPLC analysis of the phenolic compounds in the stem and leaves of Taif’s rose collected from different farms.

Ellagic acid, catechol, resorcinol, gallic acid, and phloroglucinol were the main phenolic compounds, which were separated and identified using the HPLC in Taif’s rose extract (Table 3 and Figure 1). Plants collected from F4 had the highest contents of ellagic and gallic acid (23.54, and 37.40 mg g^−1^, respectively), while those from F2 had the highest resorcinol content (18.74 mg g^−1^) in their stems. Additionally, F3 and F2 plant leaves had the highest catechol and phloroglucinol contents (21.60 and 6.24 mg g^−1^, respectively).

#### 3.3.2. Flavonoid Compounds

**Figure 2:**
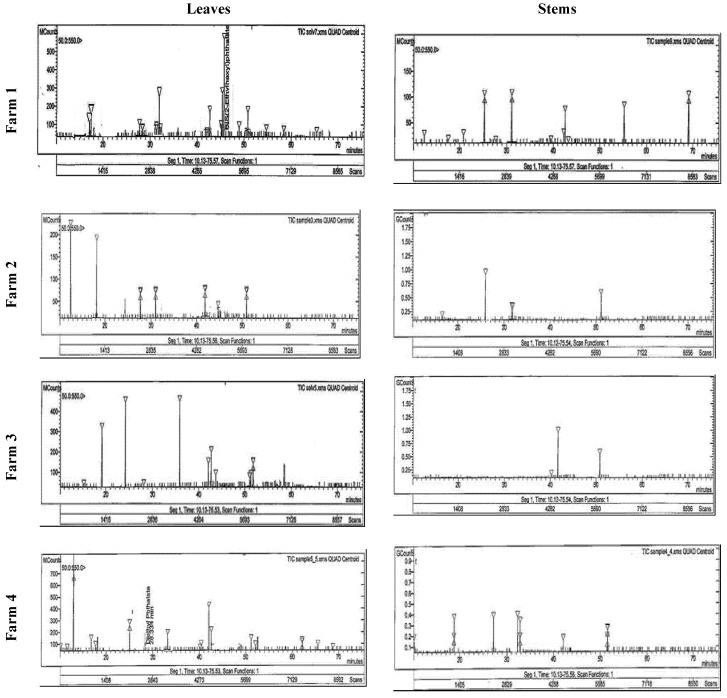
HPLC analysis of the flavonoid compounds in the stem and leaves of Taif’s rose collected from different farms.

Using HPLC, the separated and identified flavonoid compounds were apigenin, luteolin, chrysoeriol, rutin, and kaempferol (Table 4 and Figure 2). It was clear that plants had higher contents of the separated compounds (except rutin) in their stems than in their leaves. The highest contents of luteolin and chrysoeriol (30.56 and 66.20 mg g^−1^) were recorded in the stems, while the highest rutin (25.30 mg g^−1^) was recorded in the leaves of the F1 plants. In addition, the highest quercetin and apigenin (25.41 and 30.44 mg g^−1^) and kaempferol (38.74 mg g^−1^) were recorded in the stems of F4 and F3, respectively. 

#### 3.3.3. Alkaloid Compounds

**Figure 3:**
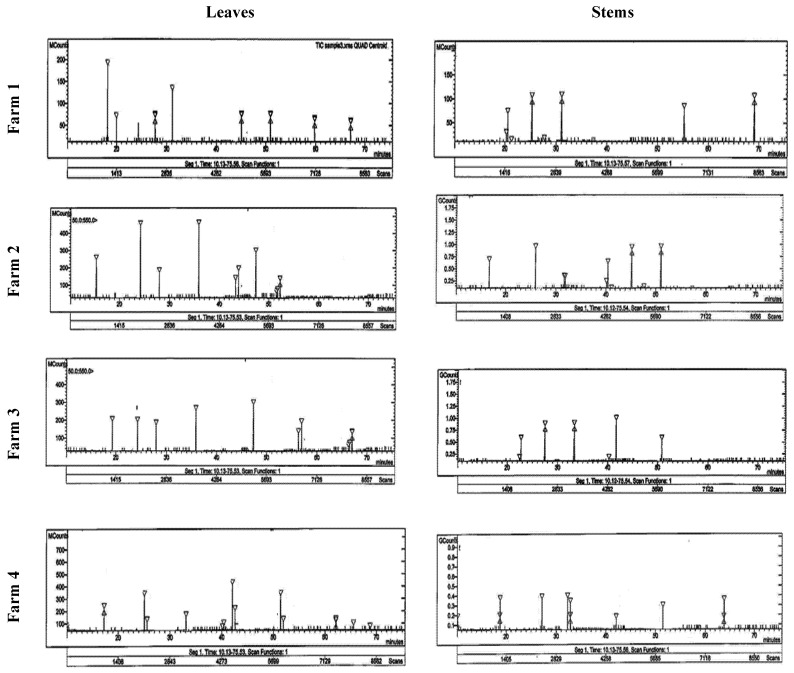
HPLC analysis of the alkaloid compounds in the stem and leaves of Taif’s rose collected from different farms.

Six alkaloid compounds (berbamine, jatrorrhizine, palmatine, reticuline, isocorydine, and boldine) were separated and identified using HPLC in Taif’s rose extract (Table 5 and Figure 3). It was found that the stems of Taif’s rose plants had higher contents of the separated compounds (except jatrorrhizine) than the leaves. The stems of the F1 plants had the highest contents of berbamine, palmatine, and isocorydine (5.24, 6.36, and 5.69 mg g^−1^, respectively), while their leaves had the highest jatrorrhizine content (9.50 mg g^−1^). In addition, the highest boldine and reticuline contents (0.89 and 8.5 mg g^−1^) were recorded in the stems of F2 and F4 plants, respectively.

### 3.4. In Vitro Antimicrobial Activity 

#### 3.4.1. Leaf Extracts

**Figure 4:**
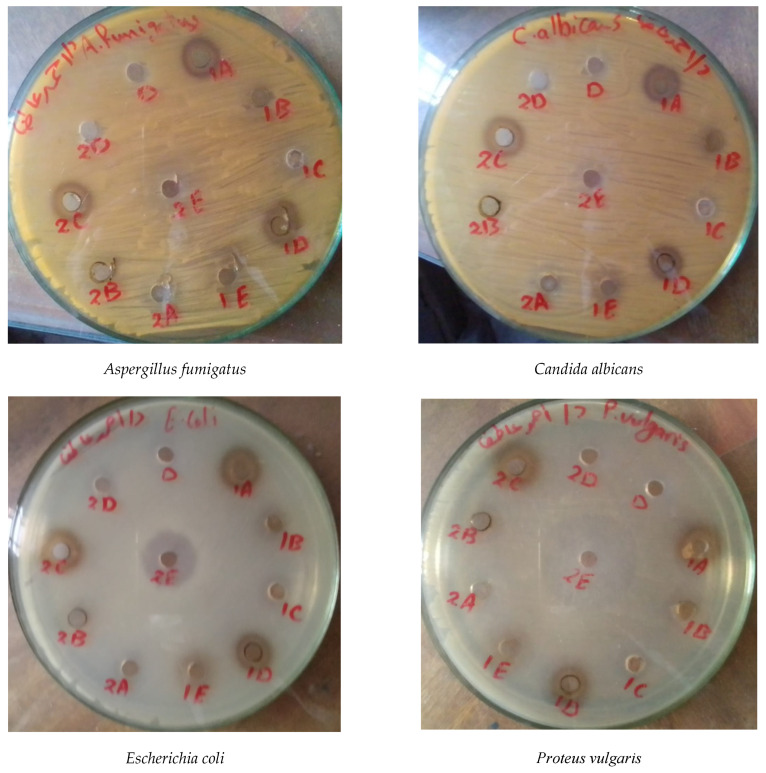

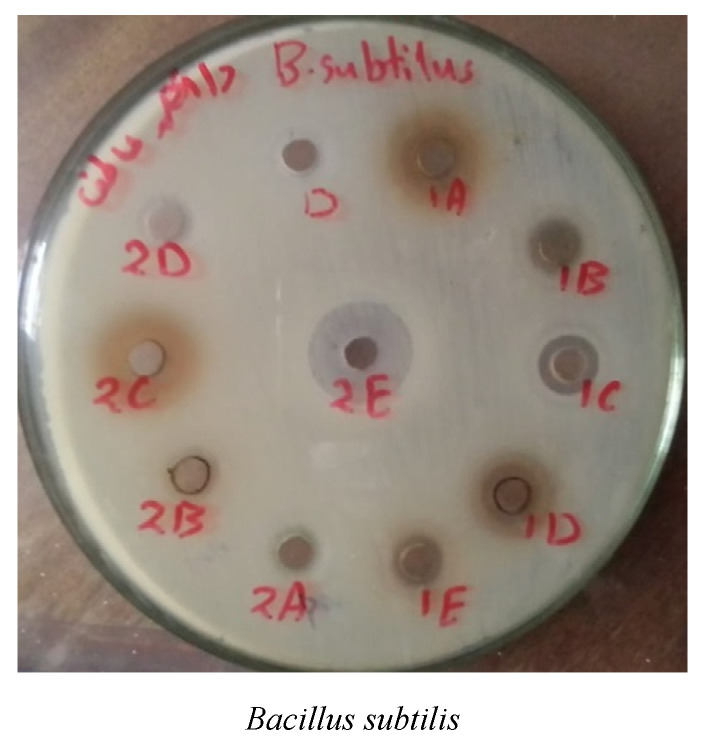
Antimicrobial activity of the different extracts of Taif’s rose. 1: stem, 2: leaf, A: methanol extract, B: ethanol extract, C: boiled water, D: cold water, E: warm water.

The pharmacological properties of Taif’s rose leaf extracts showed that the boiling water extract was exclusively active against all the studied bacterial and fungal strains, while the remaining extracts had no antifungal activities (Table 6 and Figure 4). *Bacillus subtilis, Escherichia coli*, and *Proteus vulgaris* were highly sensitive (inhibition zones = 24, 24, and 41 mm, respectively) to warm water extract compared with gentamicin (26, 30, and 17 mm). In addition, the boiling water extract was moderately active against fungal and bacterial strains with activities of 12, 10, 12, 12, and 16 mm for *Aspergillus fumigatus*, *Candida albicans, B. subtilis*, *E. coli*, and *P. vulgaris*, respectively. It is worth noting that *P. vulgaris* was exclusively sensitive to all Taif’s rose extracts as follows: warm water > ethanol > boiling water > methanol > cold water. 

#### 3.4.2. Stem Extracts

The antimicrobial activity data of the stem extracts of Taif’s rose showed that the methanol and cold water extracts were moderately active against all studied bacterial and fungal strains, while ethanol, boiling water, and warm water had no activity against fungal strains (Table 7 and Figure 4). It was clear that *P. vulgaris* was highly susceptible to all stem extracts with the highest inhibition zone (24 mm) for cold water and the lowest (13 mm) for methanol extracts. In addition, *B. subtilis* was moderately sensitive against most stem extracts (except warm water) with the highest activity (14 mm) for methanol and the lowest (11 mm) for ethanol extracts. Moreover, *A. fumigatus* and *C. albicans* were moderately susceptible to methanol (11 mm) and cold water (12 and 13 mm) extracts.

## 4. Discussion 

The pruning of Taif’s rose is a horticultural art for manipulating plant architecture to force the plant into artificial rest or a dormant period before flowering [11]. Pruning waste disposal by drying and burning or storage causes environmental pollution [28]. In the current study, the biomass of the pruning wastes ranged between 1.3 t FW ha^−1^ and 5.2 t FW ha^−1^ in F4 (youngest farm) and F3 (oldest farm), respectively. According to Al-Yasi et al. [5], approximately 860 farms with different areas in the Taif governorate and its suburbs are cultivated with Taif’s rose, which produces approximately 2730 tons of pruning waste and can cause a tremendous environmental problem. Therefore, it is of great importance and urgent need to recycle these agricultural wastes and reuse them for various economic purposes. 

Carbohydrates are energy-rich molecules that play an important role in the immune system, pathogenesis, blood clotting, fertilization, and protein folding and placement. Their determination in plants is important for quality control analysis, as they are bioinformative macromolecules [38]. It was found that Taif’s rose leaves had higher carbohydrate contents than the stems, where the contents ranged between 0.78% in the stem and 3.05% in the leaves. These values were comparable to 0.25–4.05% in the leaves of the medicinal aloe plants [39], but lower than 9.0–16.3% recorded in the leaves of *Calotropis procera* [40]. The high carbohydrate content in the leaves may be a response to the drought stress of rose plants before and after pruning [5]. In addition, the accumulation of carbohydrates may be due to the reduction in their utilization, either as a source of energy or for the formation of new cells and tissues or as an osmolyte of the cells [41]. According to Chesney and Vasquez [42], the biosynthesis of carbohydrates is influenced by pruning practices, and the stored carbohydrates can be used for plant regrowth [12].

The use of herbal/natural drugs as complementary/alternative medicines is gaining popularity throughout the world and many drugs are directly extracted from plants, whereas others are chemically modified [14]. Phytochemical exploration of the ethanolic extract of the Taif’s rose stem and leaves revealed the presence of cardiac glycosides, alkaloids, and phenolic compounds. Similar findings were reported by Fathima and Murthy [43] in the petals of the same species. According to Alzletni et al. [44], the determination of these phytochemical compounds is important to show the nutritional and medicinal value of plants. Notably, the Taif rose stem had higher cardiac glycoside and flavonoid contents, while the plant leaves had higher phenolic and alkaloid contents. According to Baydar and Baydar [7], Taif’s rose leaf extracts were rich in phenolic acids, including ferulic and gallic acids, and flavonoids, including catechin, compared to the other extracts. Cardiac glycosides are a type of secondary metabolite that has traditionally been utilized to augment cardiac contractile force in individuals suffering from cardiac arrhythmias or congestive heart failure [45]. Their contents ranged between 2.98 mg g^−1^ in the leaves of the oldest farm and 5.69 mg g^−1^ in the stems of the youngest farm. These values were lower than the 9.5–15.2 mg g^−1^ and 9.07–21.09 mg g^−1^ recorded in *C. procera* [40] and *Aloe* spp. [39], respectively. 

Plant-based secondary metabolites are known to represent several structurally diverse classes of polyphenols with potential pharmacological activities, including anticancer, anti-inflammatory, antioxidant, and antipathogenic properties [46]. The plant leaves from the oldest farm had the highest phenolic contents (12.41 mg g^−1^), while the stems of the youngest farm had the lowest (3.14 mg g^−1^). These values are lower than the 386.4 mg g^−1^ recorded in the flower residue of Taif’s rose [47] and 4.21–25.02 mg g^−1^ recorded in *Aloe* spp. [40]. This means that the pharmacological activities (antioxidant, anti-ageing, whitening, antitumor) of the flowers are greater than those of leaves and stems. According to Nayebi et al. [19], the cardioprotective effect of Taif’s rose bioactive phenolics may be attributed to the inhibition of the enzymes related to atherosclerosis and hypertension. In addition, several investigations have demonstrated the antibacterial and disinfectant activity of Taif’s rose and indicated the role of large phenolic contents such as flavonoids, terpenoids, and phenyl ethyl alcohol [20]. The highest flavonoid content (9.33 mg g^−1^) was recorded in the stems of Farm 2 plants, while the lowest (5.09 mg g^−1^) was found in the leaves of Farm 3 plants. 

Ellagic acid, catechol, resorcinol, gallic acid, and phloroglucinol were the main phenolic compounds separated and identified using HPLC in Taif’s rose extract. The content of gallic acid as the main indicator in Taif’s rose ranged between 5.6 and 37.4 mg g^−1^, which is lower than the 50.3 mg g^−1^ recorded in the flower residue of the same species [48], 7.21–40.12 mg g^−1^ recorded in *Aloe* spp. [39]. Investigations have shown that gallic acid possesses a lot of biological activities such as antioxidant properties, antimicrobial activity, anti-inflammatory, antiviral, and antimutagenic activities, and anticancer activity [49,50]. Gallic acid also has anti-biofilm activity versus *Staphylococcus aureus* [51]. Moreover, quercetin is an abundant polyphenolic flavonoid that provides many health-promoting benefits such as being potent vasodilatory agents, cancer-reducing agents, anti-inflammatory, protective against asthma, and many others [52]. Furthermore, ellagic acid is an important compound used as an anticarcinogenic, multifunctional protector against oxidative stress and an anti-inflammatory agent in the treatment of chronic ulcerative colitis [4,53,54].

HPLC analysis showed that Taif rose leaves and stems produced flavonoid compounds including luteolin, apigenin, quercetin, rutin, kaempferol, and chrysoeriol. These compounds have potential antioxidant, anti-inflammatory, and antimicrobial properties [54]. Moreover, according to Dahat et al. [55], quercetin and its glycoside rutin have been reported in extracts displaying nephroprotective properties. In addition, luteolin and apigenin have been shown to inhibit the viability of leukemic cells, colon and ovarian carcinoma cells, and particularly human breast cancer cells, as well as reduce the occurrence of mouth sores and induce mild symptomatic relief [56]. Quercetin also helps protect against certain types of cancers, especially colon cancer [49], reduces the occurrence of mouth sores, and helps to induce mild symptomatic relief [52]. Moreover, alkaloids are biologically active compounds widely used as pharmaceuticals and synthesized as secondary metabolites in plants, and many of these compounds are highly toxic [13,15]. Berbamine, jatrorrhizine, palmatine, reticuline, isocorydine, and boldine were the main alkaloid compounds in Taif’s rose. These compounds are common constituents of many Chinese medicinal plants and are known to have antibacterial, anti-inflammatory, anticancer, and choleretic properties as well as promote leukocytosis [57]. According to Duke [58], the long-term consumption of boldine led to color hallucinations, depression, partial motor aphasia, and sound hallucinations. High excitement exaggerates reflexes and respiratory movements, increases diuresis, causes cramps and convulsions, ends in death from centric respiratory paralysis, and heartbeats sometimes fail after respiration.

The antimicrobial activity of a plant depends on the phytogeographical area, the plant part, and the extraction process [59]. The pharmacological properties of Taif’s rose leaf extracts showed that the boiling water extract was moderately active against all studied bacterial and fungal strains, and the remaining extracts had no antifungal activities. Gram-positive (*B. subtilis*), and gram-negative (*E. coli*, and *P. vulgaris)* bacteria were highly sensitive to warm water extracts compared with gentamicin antibiotics (control). In a similar study on *Rosa indica* extracts, Saeed et al. [60] found antibacterial activity against *Proteus* sp. and *E. coli*, and antifungal activity against *A. fumigatus* strains. The most effective reason is the presence of various phytochemical compounds such as alkaloids, phenolic acids, flavonoids, tannins, and other phenolic compounds. According to Baydar and Baydar [7], the total phenolics were higher in the cold and hot extractions of the leaf. In addition, the total phenolic and flavonoid contents have a good correlation with antioxidant activity [61], which is an important factor in assessing the biological activity of medicinal plants in the rose species [62]. Moreover, Samuelsen [63] and Abd Razik et al. [64] attributed the antimicrobial activity to the presence of some intermediately polar or nonpolar substances of relatively low molecular weight in the plant extract. 

The methanol and cold water extracts of the stem were active against all studied bacterial and fungal strains; however, the remaining extracts had no activity against fungal strains. Similar findings reported that the methanol extract of *P. major* showed higher antimicrobial activity than the ethanol extract [55,65]. According to Norziah et al. [66], the use of water as the extracting solvent is more desirable than the use of organic solvents due to its environmentally friendly and non-toxic characteristics. Moreover, water is a good solvent in extracting a considerable quantity of phenolic and flavonoid compounds with high activities that can safely be exploited in numerous food applications. In one study, the intraperitoneal administration of 10 mg kg^−1^ of *R. damascena* Mill. methanolic extract in infected mice significantly reduced the parasitemia of *Plasmodium berghei* [19]. It was clear that *P. vulgaris* was highly susceptible, while *B. subtilis* was moderately sensitive against all stem extracts. Similar results were reported by Halawani [67] on the different extracts of *R. damascena*. Conversely, the aqueous extract of the medicinal plant *P. major* has no antimicrobial activity [55]. Therefore, pharmaceutical studies are required to separate, purify, and identify the phytochemical compounds in the ethanolic, methanolic, and water extracts of the pruning wastes of Taif’s rose. In addition, the antibacterial activity of each compound was investigated to determine the compound/s that has antibacterial activity against pathogenic microorganisms.

## 5. Conclusions

Processing waste materials (such as rose wastes) and converting them into useful and efficient materials is an important issue that needs more consideration. The current study revealed that the pruning wastes of Taif’s rose could be recycled due to their biologically active compounds including alkaloids, flavonoids, and phenolic compounds. More than 2700 tons of pruning waste is produced annually from approximately 860 rose farms in Taif Province. The phytochemical screening of Taif’s rose indicated the presence of a considerable content of cardiac glycosides, which have traditionally been utilized to augment cardiac contractile force in individuals suffering from cardiac arrhythmias or congestive heart failure. HPLC analysis showed that Taif’s rose contains flavonoid compounds including luteolin, apigenin, quercetin, rutin, kaempferol, and chrysoeriol; phenolics including ellagic acid, catechol, resorcinol, gallic acid, and phloroglucinol; alkaloids including berbamine, jatrorrhizine, palmatine, reticuline, isocorydine, and boldine. These compounds have several pharmacological properties including antimicrobial activities. Ethanol and methanol extracts of Taif’s rose showed antimicrobial activity, but the highest was found in the water extracts. Further studies on the phytochemical constituents and pharmacological activity of the distillation wastes of Taif rose are currently underway.

## Figures and Tables

**Table 1:** Fresh biomass (mean: upper line, SD: lower line) of the vegetative wastes produced after pruning of four Taif’s rose farms on the Al-Shafa highland.

Farm	Farm 1	Farm 2	Farm 3	Farm 4	F-Value
Mean	2.5c	3.7b	5.2a	1.3d	128.5 ***
SD	0.4	0.5	0.6	0.4	

Means with the same letter in the same row are not significantly different (Duncan’s multiple range tests at *p* < 0.05), ***: meam *p* < 0.001).

**Table 2:** Phytochemical constituents (mean ± SD) of the leaves and stems of Taif’s rose collected from different rose farms. Maximum and minimum values are underlined.

Farm		Carbohydrates %	Cardiac Glycosides mg Securiaside g^−1^	Phenolics mg GAE g^−1^	Flavonoids mg RUE g^−1^	Alkaloids mg AE g^−1^
Farm 1	L	1.26±0.05d	4.33±0.23c	10.26±1.01b	5.33±0.62g	10.96±1.32a
	S	0.78±0.10g	4.33±0.20c	7.22±0.87e	7.11±1.02c	7.12±0.94d
Farm 2	L	1.69±0.04c	3.97±0.22e	8.36±1.32c	6.14±1.04f	8.36±2.08c
	S	0.96±0.10e	5.14±0.19b	6.07±0.71f	9.33±2.13a	8.36±1.62c
Farm 3	L	3.05±0.05a	2.98±0.32g	12.41±2.13a	5.09±0.92h	10.07±2.12b
	S	0.88±0.12f	4.23±0.26d	5.36±1.06g	7.01±0.82d	4.09±0.26e
Farm 4	L	2.36±0.09b	3.14±0.41f	8.19±1.04d	8.76±1.63b	8.36±1.27c
	S	0.79±0.14g	5.69±0.72a	3.14±0.23h	6.39±0.05e	3.22±0.43f
F-value		2346.5 ***	2839.7 ***	1929.4 ***	3980.4 ***	1038.5 ***

L: leaves, S: stem, GAE: gallic acid equivalent, RUE: rutin equivalent, AE: atropine equivalent. Means with the same letter in the same row are not significantly different (Duncan’s multiple range tests at *p* < 0.05), ***: meam *p* < 0.001).

**Table 3:** HPLC analysis of the phenolic concentration of the leaves and stems of Taif’s rose collected from different rose farms. ND: not detected.

Farm		Phenolics Concentration (mg g^−1^)				
		Gallic Acid	Ellagic Acid	Catechol	Resorcinol	Phloroglucinol
Farm 1	L	5.60	ND	23.54	34.20	ND
	S	9.45	ND	13.44	18.74	0.96
Farm 2	L	19.58	14.50	3.54	0.25	6.24
	S	33.60	19.58	2.66	1.23	ND
Farm 3	L	15.63	ND	21.60	4.12	ND
	S	13.65	1.58	6.28	ND	0.89
Farm 4	L	16.04	1.38	6.11	9.05	ND
	S	37.40	23.54	3.96	2.74	ND

L: leaves, S: stem.

**Table 4:** HPLC analysis of the flavonoid concentration of the leaves and stems of Taif’s rose collected from different rose farms. ND: not detected.

Farm		Flavonoids Concentration (mg g^−1^)					
		Quercetin	Apigenin	Luteolin	Chrysoeriol	Rutin	Kaempferol
Farm 1	L	18.77	2.6	1.99	ND	25.3	ND
	S	0.87	17.22	30.56	66.2	ND	18.32
Farm 2	L	12.87	6.58	10.5	ND	19.85	21.68
	S	17.02	ND	25.6	44.05	3.8	38.74
Farm 3	L	1.63	ND	18.02	19.2	6.74	ND
	S	7.15	ND	22.6	14.8	ND	19.23
Farm 4	L	ND	ND	ND	ND	15.6	21.5
	S	25.41	30.44	5.6	55.69	18.52	33.91

L: leaves, S: stem.

**Table 5:** HPLC analysis of the alkaloid concentration of the leaves and stems of Taif’s rose collected from different rose farms. ND: not detected.

Farm		Alkaloid’s Concentration (mg g^−1^)					
		Berbamine	Jatrorrhizine	Palmatine	Reticuline	Isocorydine	Boldine
Farm 1	L	3.14	0.69	ND	2.36	ND	ND
	S	5.24	7.13	6.36	ND	5.69	ND
Farm 2	L	4.69	9.5	ND	ND	1.6	N.D
	S	ND	ND	ND	ND	3.7	0.89
Farm 3	L	1.25	ND	ND	ND	ND	ND
	S	ND	ND	2.45	ND	ND	0.39
Farm 4	L	ND	ND	ND	1.4	ND	ND
	S	ND	4.44	1.69	8.5	ND	ND

L: leaves, S: stem.

**Table 6:** Antimicrobial activity (mm) of the different extracts of Taif’s rose leaves on the pathogenic bacterial and fungal strains. NA: no activity.

Extract	Fungi		Bacteria		
			Gram-Positive	Gram-Negative	
	*Aspergillus fumigatus*	*Candida albicans*	*Bacillus subtilis*	*Escherichia coli*	*Proteus vulgaris*
Control	Ketoconazole		Gentamicin	Gentamicin	
	17	20	26	30	17
Methanol	NA	NA	NA	NA	12
Ethanol	NA	NA	NA	NA	17
Boiling Water	12	10	12	12	16
Cold Water	NA	NA	10	NA	11
Warm Water	NA	NA	24	24	41

**Table 7:** Antimicrobial activity (mm) of the different extracts of Taif’s rose stem on the pathogenic bacterial and fungal strains. NA: no activity.

Extract	Fungi		Bacteria		
			Gram-Positive	Gram-Negative	
	*Aspergillus fumigatus*	*Candida albicans*	*Bacillus subtilis*	*Escherichia coli*	*Proteus vulgaris*
Control	Ketoconazole		Gentamicin	Gentamicin	
	17	20	26	30	17
Methanol	11	11	14	13	13
Ethanol	NA	NA	11	NA	18
Boiling Water	NA	NA	12	NA	15
Cold Water	12	13	12	14	24
Warm Water	NA	NA	NA	NA	20

## Data Availability

Not applicable.
